# Psychosocial and Behavioral Risk Profiles of Cigarette Smokers and E-Cigarette Users Among Adolescents in Minnesota: The 2016 Minnesota Student Survey

**DOI:** 10.5888/pcd15.180222

**Published:** 2018-09-27

**Authors:** Tara E. Jenson

**Affiliations:** 1 Milken Institute School of Public Health, The George Washington University, Washington, DC

## Abstract

**Introduction:**

Understanding differences in predictors of adolescent cigarette smoking and e-cigarette use can inform public health strategies for preventing and reducing tobacco use among this population. The objective of this study was to examine the association of socioeconomic, psychosocial, and behavioral factors with cigarette smoking and e-cigarette use among adolescents in Minnesota.

**Methods:**

Records (n = 126,868) were used from the 2016 Minnesota Student Survey for prevalence of and factors associated with cigarette smoking and e-cigarette use among students in grades 8, 9, and 11. Logistic regression models were used to estimate risk for smoking cigarettes, using e-cigarettes, or concurrent use of both for key independent variables.

**Results:**

American Indian students were 3.6 times as likely to report smoking cigarettes (OR = 3.57; 95% CI, 3.04–4.19), and 1.7 times as likely to report using e-cigarettes (OR = 1.72; 95% CI, 1.47–2.01) as non-Hispanic white students. Bisexual students were 4 times as likely (adjusted odds ratio [AOR] = 4.40; 95% confidence interval [CI], 4.01–4.82) as heterosexual students to smoke cigarettes and twice as likely (AOR = 2.24; 95% CI, 2.06–2.43) to use e-cigarettes. Students receiving free/reduced lunch were nearly twice as likely (AOR = 1.92; 95% CI, 1.80–2.05) to smoke cigarettes and 1.3 times as likely (AOR = 1.33; 95% CI, 1.27–1.39) to use e-cigarettes. Increasing alcohol use and decreasing academic performance were associated with increasing likelihood of cigarette smoking and e-cigarette use, more so with cigarette smoking.

**Conclusion:**

Results expand on existing research that show differences in psychosocial and behavioral risk factors between adolescent cigarette smokers and adolescent e-cigarette users.

## Introduction

E-cigarettes are a type of noncombustible tobacco product designed to allow inhalation of nicotine via vaporization of a nicotine-containing solution (1). Although the health risks of cigarette smoking are well established (2), those of e-cigarettes are largely unknown because e-cigarettes have emerged as a commercially available product in the United States only since 2007 (3). However, both the Centers for Disease Control and Prevention and the Minnesota Department of Health assert that e-cigarette use among adolescents is a health concern (4,5). Rates of cigarette smoking among high school students have trended downward nationally from 15.8% in 2011 to 8.0% in 2016 (6) and in the state of Minnesota among students in grade 9 from 19.6% in 2001 to 4.3% in 2016 (5). However, the rates of e-cigarette use among high school students overall have trended upward nationally, from 1.5% in 2011 to 11.3% in 2016, and as of 2016 was at 17.1% among 11th-grade students in Minnesota (5,6).

A study in 2017 showed that prediction-model factors associated with cigarette smoking differed significantly from factors associated with e-cigarette use among adolescents in the United States (3). Regression models of key psychosocial factors that predicted the risk of adolescents becoming cigarette smokers approximately 75% of the time predicted adolescents becoming e-cigarette smokers only approximately 25% of the time.

Further understanding of potential differences in behavioral factors and other predictors of cigarette smoking and e-cigarette use among adolescents is critical to informing comprehensive public health strategies targeting prevention and reduction of tobacco use among this population. The objective of this study was to describe the association of key socioeconomic, psychosocial, and behavioral factors with cigarette smoking and e-cigarette use among adolescents in Minnesota.

## Methods

Data for this study were sourced entirely from the 2016 Minnesota Student Survey (MSS) data set. The MSS is an anonymous, school-based, cross-sectional survey developed by 4 state agencies: the Department of Education, the Department of Health, the Department of Human Services, and the Department of Public Safety. The survey is administered every 3 years by local school districts (7). Data for the 2016 MSS were provided by public school students in Minnesota via local public school districts, and the data set is managed by the Minnesota Student Survey Interagency Team 2016 (8). The 2016 MSS data set contains 287 variables generated from approximately 112 questions on substance use, sexuality, academic performance, and other health and lifestyle behaviors and factors. The 2016 MSS is representative of 85% of Minnesota school districts (282 of 330) and comprises completed surveys of 168,733 Minnesota public school students across grades 5, 8, 9, and 11 (9). Some questions on the 2016 MSS, including those relating to the use of alcohol, drugs, and tobacco are asked only of students in grades 8, 9, and 11. Inquiry into sexual identity is asked only of students in grades 9 and 11. Because this study focused on cigarette smoking and e-cigarette use, data were analyzed for students in grades 8, 9, and 11 only. Records with missing values for demographic characteristics, targeted psychosocial and behavioral-related questions, or questions on use of cigarettes, e-cigarettes, other tobacco products, or marijuana were excluded from analyses of those topics. After excluding data for students in grade 5, the total number of records analyzed for this study was 126,868. The average age of respondents was 14.8 years (standard deviation, 1.3 y; range, 12 to 19–20 y [categories were whole numbers 12–18, then age 19–20]). Approval for use of the 2016 MSS data set was provided by the Minnesota Student Interagency Team (8) after institutional review board approval from the George Washington University Committee on Human Research.

### Variables

For this study, 3 dependent outcome variables were created: current cigarette smokers, current e-cigarette users, and concurrent cigarette smokers and e-cigarette users. Students were categorized as current cigarette smokers if they indicated they had smoked cigarettes at least 1 day in the past 30 days by making any selection other than “0 days” to the question “During the last 30 days, on how many days did you smoke a cigarette?” Students were categorized as current e-cigarette users if they indicated they had used e-cigarettes at least 1 day in the past 30 days by making any selection other than “0 days” to the question “During the last 30 days, on how many days did you use an electronic cigarette (e-cigarette, e-hookah, vaping pen)?” Students were categorized as concurrent cigarette smokers and e-cigarette users if they indicated they had both smoked cigarettes and used e-cigarettes at least 1 day in the past 30 days by making any selection other than “0 days” to both questions on cigarette smoking and e-cigarette use.

Grade level (8, 9, or 11), sex (male or female), and race/ethnicity were assessed for baseline association with cigarette smoking and e-cigarette use outcomes. For race/ethnicity, students were asked 3 questions: “Are you Hispanic or Latino(a)?,” “Are you Somali?,” and “Are you Hmong?” Respondents were allowed to respond yes or no or leave blank (categorized as no response). Additionally, students were asked, “What is your race?” and were allowed to choose 1 or more of the following (or leave blank): American Indian or Alaskan Native, Asian, black, African, or African American, Native Hawaiian or other Pacific Islander, or white. The MSS Interagency Team then compiled these responses into the combined variable of race/ethnicity with the following categories: American Indian non-Hispanic, Asian non-Hispanic, black non-Hispanic, Pacific Islander non-Hispanic, white non-Hispanic, multiple races non-Hispanic, Hispanic, and race/ethnicity missing. Grade, sex, and race/ethnicity were then adjusted for in subsequent analysis of other independent variables because of the potential of these 3 variables as confounders that might be associated with differing cigarette and e-cigarette usage patterns, familiarity, and/or cultural norms. Only the race/ethnicity variable was used in controlling for race/ethnicity in subsequent analysis. Because of large numbers of missing responses to the questions on Hmong and Somali ethnicity, these 2 variables were excluded from the regression analysis.

The following independent socioeconomic, psychosocial, and behavioral indicator variables were analyzed for association with cigarette smoking and e-cigarette use outcomes: sexual identity (heterosexual, bisexual, gay/lesbian, not sure, or questioning), economic hardship (whether students receive free or reduced-price lunch and whether students skipped meals in the past 30 days because their family did not have enough money for food) , alcohol use in the past 30 days (0 days, 1 or 2 days, 3–5 days, 6–9 days, 10–19 days, 20–29 days, all 30 days), and academic performance (mostly As, mostly Bs, mostly Cs, mostly Ds, mostly Fs, mostly incompletes, or none of these letter grades).

### Statistical analysis

Frequency analysis was conducted on the dependent variables of current cigarette smoking, e-cigarette use, and concurrent use of both as well as demographic categorical independent variables. To assess for significant association at a .05 level between each dichotomous dependent variable and the demographic, socioeconomic, psychosocial, and behavioral categorical independent variables, χ^2^ and Fisher exact test bivariate analyses were conducted. 

Frequency and bivariate analyses were conducted by using IBM SPSS Statistics for Macintosh, version 24.0 (IBM Corporation); SAS software (SAS Institute Inc) surveylogistic method was used to conduct multivariate logistical regression analysis for generating odds ratios for risk of smoking cigarettes, e-cigarette use, and concurrent use of both. All independent variables included were demonstrated to be significant at a .05 level in bivariate analyses for association with smoking cigarettes, e-cigarette use, and concurrent use of both. Regression models that included grade, sex, and race/ethnicity only were used to generate baseline odds ratios for cigarette smoking, e-cigarette use, and concurrent use of both. Separate regression models for sexual identity, socioeconomic indicators, academic performance, and alcohol use, each controlling for grade, sex, and race/ethnicity were then used to generate adjusted odds ratios for cigarette smoking, e-cigarette use, and concurrent use of both. Final determination of independent variables used in regression analysis was guided by forward selection as described previously (10) in conjunction with my own interests in factors for investigation.

Small amounts of data were missing from analysis, where no answer was provided by the respondent for one or more control variables of grade, sex, and race/ethnicity, independent variables, and outcome variables. All observations for grade level contained a response. Missing data was less than 1% for sex (n = 373) and race/ethnicity (n = 1,047), 1.2% (n = 1,521) for the question on free or reduced-price lunch, 2.9% (n = 3,714) for the question on skipped meals, 1.6% (n = 1,267) for the question on sexual identity, 4.9% (n = 6,213) for the question on Somali race/ethnicity, 4.6% (n =6,170) for the question on Hmong race/ethnicity, 6.9% (n = 8,763) for the question on alcohol use, 6.9% (n = 8,760) for the question on e-cigarette use, and the 7.0% (n = 8,850) for question on cigarette smoking.

## Results

The distribution of female adolescents and male adolescents was similar across grades; of 126,868 students, 49.5% were female, and 50.5% were male (Table 1). Representation of students in grade 8 (35.5%) and grade 9 (35.7%) was similar, whereas 28.8% of students were in grade 11. Across all 3 grades, 1.2% of respondents were American Indian non-Hispanic, 6.0% Asian non-Hispanic, 6.3% black non-Hispanic, 7.4% multiracial, 0.2% Pacific Islander non-Hispanic, and 68.6% white non-Hispanic; 9.5% of respondents were Hispanic, 1.9% Somali, and 2.9% Hmong.

**Table 1:** Prevalence of Cigarette Smoking and E-Cigarette Use in the Past 30 Days, by Selected Demographic, Socioeconomic and Behavioral Characteristics Among Adolescents in Minnesota (N = 126,868), Minnesota Student Survey, 2016^a^

Variable	Total, No. (%) (N = 126,868)	Cigarettes, No. (%) (n = 5,816)	E-Cigarettes, No. (%) (n = 12,101)	Both Cigarettes and E-Cigarettes, No. (%) (n =4,015)
**Grade level**				
Grade 8	44,983 (35.5)	1,176 (20.2)	2,412 (19.9)	776 (19.3)
Grade 9	45,309 (35.7)	1,787 (30.7)	3,891 (32.2)	1,236 (30.8)
Grade 11	35,576 (28.8)	2,853 (49.1)	5,798 (47.9)	2,003 (49.9)
**Sex**				
Male	63,818 (50.5)	2,738 (47.1)	6,328 (52.3)	1,976 (49.2)
Female	62,677 (49.5)	3,064 (52.7)	5,747 (47.5)	2,030 (50.6)
**Race/ethnicity^b^**				
American Indian	1,516 (1.2)	188 (3.2)	203 (1.7)	104 (2.6)
Asian	7,551 (6.0)	128 (2.2)	361 (3.0)	89 (2.2)
Black	8,052 (6.3)	213 (3.7)	488 (4.0)	149 (3.7)
Hispanic	12,040 (9.5)	652 (11.2)	1,402 (11.6)	451 (11.2)
Multiple races	9,372 (7.4)	640 (11.0)	1,196 (9.9)	439 (10.9)
Pacific Islander	207 (0.2)	14 (0.2)	25 (0.2)	11 (0.3)
White	87,083 (68.6)	3,949 (67.9)	8,351 (69.0)	2,747 (68.4)
Somali^c^	2,406 (1.9)	88 (1.5)	137 (1.1)	71 (1.8)
Hmong^c^	3,631 (2.9)	92 (1.6)	213 (1.8)	65 (1.6)
**Sexual identity^d^**				
Heterosexual	72,305 (89.7)	3,506 (76.3)	8,256 (85.9)	2,462 (76.7)
Bisexual	4,014 (5.0)	727 (15.8)	860 (9.0)	502 (15.6)
Gay or lesbian	1,027 (1.3)	128 (2.8)	167 (1.7)	85 (2.7)
Not sure or questioning	3,272 (4.1)	233 (5.1)	324 (3.4)	160 (5.0)
**Economic status**				
Receives free or reduced-price lunch	35,663 (28.5)	2,344 (40.5)	3906 (32.4)	1564 (39.2)
In past 30 days, skipped meals because family did not have enough money to buy food	5,700 (4.6)	866 (15.0)	1,269 (10.5)	620 (15.6)
**Grades reported^e^**				
Mostly As	54914 (43.8)	763 (13.3)	2,515 (21.1)	484 (12.2)
Mostly Bs	43,702 (34.9)	1,910 (33.3)	4,576 (38.3)	1,307 (33.0)
Mostly Cs	19,125 (15.3)	1,865 (32.5)	3,245 (27.2)	1,330 (33.5)
Mostly Ds	4,483 (3.6)	682 (11.9)	982 (8.2)	486 (12.3)
Mostly Fs	1,716 (1.4)	354 (6.2)	420 (3.52)	238 (6.0)
**No. of days of alcohol use in past 30 days**				
0	101,737 (86.1)	1,777 (30.8)	4,946 (41.2)	1,010 (25.4)
1 or 2	9,931 (8.4)	1,612 (28.0)	3,416 (28.5)	1,096 (27.5)
3–5	3,519 (3.0)	1,062 (18.4)	1,838 (15.3)	824 (20.7)
6–9	1,613 (1.4)	663 (11.5)	1,002 (8.4)	522 (13.1)
10–19	811 (0.7)	441 (7.7)	537 (4.5)	357 (9.0)
20–29	205 (0.2)	111 (1.9)	129 (1.1)	90 (2.3)
All 30	289 (0.2)	102 (1.8)	130 (1.1)	83 (2.1)

a Numbers do not total expected value because of missing data. Percentages are based on n’s in column head.

b All categories are non-Hispanic, except Hispanic.

c Somali and Hmong race/ethnicity asked about as a yes-or-no question separately from the question on race.

d Sexual identity inquired of grades 9 and 11 only.

e Numbers may not total expected value because of a small number of responses of “mostly incompletes” or “none of these letter grades.”

Of 126,868 students, 13,902 (11.0%) reported smoking cigarettes or using e-cigarettes in the past 30 days: 5,816 students (4.6%) reported smoking cigarettes on at least 1 day, 12,101 students (9.5%) reported using e-cigarettes on at least 1 day, 1,801 students (1.4%) reported smoking cigarettes only, 8,086 (6.4%) reported using e-cigarettes only, and 4,015 (3.2%) reported both smoking cigarettes and using e-cigarettes in the past 30 days.

Logistic regression analysis of grade, sex, and race/ethnicity in relation to cigarette smoking, e-cigarette use, and concurrent use of cigarettes and e-cigarettes demonstrated significant association for grade and most races/ethnicities but not for sex (Table 2). Students in grade 11 were 3.5 times as likely to report using e-cigarettes (OR = 3.50; 95% confidence interval [CI], 3.33–3.68) and 3.3 times as likely to report smoking cigarettes (OR = 3.34; 95% CI, 3.11–3.58) in the past 30 days as students in grade 8. American Indian students were 3.6 times as likely to report smoking cigarettes (OR = 3.57; 95% CI, 3.04–4.19), and 1.7 times as likely to report using e-cigarettes (OR = 1.72; 95% CI, 1.47–2.01) as non-Hispanic white students. Asian students were 0.33 times as likely to report smoking cigarettes (OR = 0.33; 95% CI, 0.26–0.41), and 0.47 times as likely to report using e-cigarettes (OR = 0.47; 95% CI, 0.41–0.54) as non-Hispanic white students. 

**Table 2:** Crude Odds Ratios for Cigarette Smoking and E-Cigarette Use in the Past 30 Days, by Selected Demographic Characteristics Among Adolescents in Minnesota (N = 126,868), Minnesota Student Survey, 2016

Demographic Characteristic	Cigarettes		E-cigarettes		Both Cigarettes and E-Cigarettes	
	Crude OR^a^ (95% CI)	*P* Value^b^	Crude OR^a^ (95% CI)	*P* Value^b^	Crude OR^a^ (95% CI)	*P* Value^b^
**Grade level**						
Grade 8	1.0 [Reference]	NA	1.0 [Reference]	NA	1.0 [Reference]	NA
Grade 9	1.58 (1.46–1.70)	<.001	1.71 (1.62–1.80)	<.001	1.64 (1.50–1.80)	<.001
Grade 11	3.34 (3.11–3.58)	<.001	3.50 (3.33–3.68)	<.001	3.47 (3.19–3.77)	<.001
**Sex^c^**						
Male	1.0 [Reference]	NA	1.0 [Reference]	NA	1.0 [Reference]	NA
Female	1.11 (1.01–1.17)	.80	0.88 (0.84–0.91)	.42	1.01 (0.95–1.08)	.97
**Race/ethnicity^d^**						
American Indian	3.57 (3.04–4.19)	<.001	1.72 (1.47–2.01)	<.001	2.65 (2.15–3.26)	<.001
Asian	0.33 (0.26–0.41)	<.001	0.47 (0.41–0.54)	<.001	0.33 (0.25–0.43)	<.001
Black	0.67 (0.57–0.72)	<.001	0.81 (0.73–0.91)	<.001	0.64 (0.52–0.78)	<.001
Hispanic	1.37 (1.25–1.49)	.001	1.45 (1.37–1.55)	<.001	1.36 (1.23–1.51)	.003
Multiple races	1.74 (1.60–1.90)	<.001	1.59 (1.49–1.70)	<.001	1.70 (1.53–1.89)	<.001
Pacific Islander	1.72 (0.98–3.01)	.09	1.47 (0.94–2.30)	.15	1.94 (1.03–3.63)	.05
White	1.0 [Reference]	NA	1.0 [Reference]	NA	1.0 [Reference]	NA
Somali^e^	1.40 (1.07–1.83)	.007	0.93 (0.76–1.15)	.43	1.68 (1.24–2.28)	<.001
Hmong^e^	1.43 (1.07–1.90)	.09	1.15 (0.95–1.40	.14	1.47 (1.05–2.05)	.14

Abbreviations: CI, confidence interval; NA, not applicable; OR, odds ratio.

a Crude ORs calculated by using a logistic regression model for grade, sex, and race/ethnicity.

b Significant at *P* = .05.

c Numbers do not total expected value because of a small portion of responses of “no answer” to the question, “what is your biological sex?”

d All categories are non-Hispanic, except Hispanic. Percentages for racial or ethnic categories of American Indian, Asian, black, Hispanic, multiple races, Pacific Islander, and white do not total 100 because of a small portion of responses of “no answer.”

e Somali and Hmong race/ethnicity asked about as a yes-or-no question separately from the question on race; reference group for each is “no response.”

Logistic regression analysis of sexual identity in relation to cigarette smoking, e-cigarette use, and concurrent use of both overall demonstrated significant association, except for gay/lesbian respondents and e-cigarette use (Table 3). Bisexual students were more than 4 times as likely (adjusted OR [AOR] = 4.40; 95% CI, 4.01–4.82) as heterosexual students to smoke cigarettes but only twice as likely (AOR = 2.24; 95% CI, 2.06–2.43) to use e-cigarettes.

**Table 3:** Adjusted Odds Ratios for Cigarette Smoking and E-Cigarette Use in the Past 30 Days, by Selected Socioeconomic and Behavioral Characteristics Among Adolescents in Minnesota (N = 126,868), Minnesota Student Survey, 2016

Risk Factor	Cigarettes		E-Cigarettes		Both Cigarettes and E-Cigarettes	
	Adjusted OR^a^ (95% CI)	*P* Value^b^	Adjusted OR^a^ (95% CI)	*P* Value^b^	Adjusted OR^a^ (95% CI)	*P* Value^b^
**Sexual identity^c^**						
Heterosexual (straight)	1.0 [Reference]	NA	1.0 [Reference]	NA	1.0 [Reference]	NA
Bisexual	4.40 (4.01–4.82)	<.001	2.24 (2.06–2.43)	<.001	4.22 (3.79–4.69)	<.001
Gay/lesbian	2.75 (2.27–3.34)	.001	1.48 (1.24–1.76)	.19	2.52 (2.00–3.17)	.03
**Economic status^d^**						
Receives free or reduced-price lunch	1.92 (1.80–2.05)	<.001	1.33 (1.27–1.39)	<.001	1.77 (1.64–1.91)	<.001
In last 30 days, skipped meals because family did not have enough money to buy food	3.63 (3.33–3.95)	<.001	2.79 (2.59–2.99)	<.001	3.70 (3.35–4.08)	<.001
**Grades reported**						
Mostly As	1.0 [Reference]	NA	1.0 [Reference]	NA	1.0 [Reference]	NA
Mostly Bs	2.47 (2.25–2.70)	<.001	1.91 (1.80–2.01)	<.001	2.63 (2.35–2.94)	<.001
Mostly Cs	4.58 (4.14–5.07)	<.001	2.75 (2.58–2.94)	<.001	4.93 (4.36–5.58)	<.001
Mostly Ds	5.89 (5.16–6.72)	<.001	3.10 (2.81–3.41)	<.001	6.11 (5.24–7.14)	<.001
Mostly Fs	8.08 (6.81–9.59)	<.001	3.64 (3.16–4.19)	<.001	7.42 (6.08–9.06)	<.001
**No. of days of alcohol use in past 30 days**						
0	1.0 [Reference]	NA	1.0 [Reference]	NA	1.0 [Reference]	NA
1 or 2	9.79 (9.08–10.6)	<.001	9.25 (8.78–9.75)	<.001	11.3 (10.3–12.4)	<.001
3–5	21.5 (19.6–23.5)	.01	18.7 (17.3–20.1)	<.001	27.4 (24.7–30.5)	<.001
6–9	34.5 (30.7–38.7)	<.001	27.5 (24.7–30.7)	<.001	42.5 (37.3–48.3)	<.001
10–19	61.6 (52.9–71.7)	<.001	34.4 (29.5–40.2)	<.001	72.0 (61.4–84.3)	<.001
20–29	68.4 (50.8–92.0)	<.001	34.3 (25.2–46.8)	<.001	78.7 (58.5–106.0)	<.001
All 30	31.2 (24.0–40.6)	<.001	15.2 (11.8–19.7)	.33	40.0 (30.3–52.7)	<.001

Abbreviations: CI, confidence interval; NA, not applicable; OR, odds ratio.

a Adjusted ORs calculated by using a logistic regression model adjusted for grade, sex, and race/ethnicity.

b Significant at *P* = .05.

c Sexual identity inquired of grades 9 and 11 only.

d Reference group is students who responded no.

Logistic regression analysis of students who reported receiving free or reduced-price lunch at school or skipping meals because of economic hardship was significantly associated with increased likelihood of cigarette smoking, e-cigarette use, and concurrent use of both (Table 3). Students receiving free or reduced-price lunch were nearly twice as likely (AOR = 1.92; 95% CI, 1.80–2.05) to smoke cigarettes but only 1.33 times as likely (AOR = 1.33; 95% CI, 1.27–1.39) to use e-cigarettes as students not receiving such lunch. Students reporting skipping meals were more than 3.5 times as likely (AOR = 3.63; 95% CI, 3.33–3.95) to smoke cigarettes but only 2.79 times as likely (AOR = 2.79; 95% CI, 2.59–2.99) to use e-cigarettes as students not skipping meals.

Logistic regression analysis demonstrated significant association between academic performance and cigarette smoking, e-cigarette use, and concurrent use of both (Table 3). Students reporting mostly Bs were more than twice as likely (AOR = 2.47; 95% CI, 2.25–2.7) to have smoked cigarettes in the past 30 days and nearly twice as likely (AOR = 1.91; 95% CI, 1.80–2.01) to have used e-cigarettes in the past 30 days as students reporting that they receive mostly As. Students reporting mostly Fs were 8 times as likely (AOR = 8.08; 95% CI, 6.81–9.59) to smoke cigarettes but only 3.64 times as likely (AOR = 3.64; 95% CI, 3.16–4.19) to use e-cigarettes as students reporting mostly As. 

Logistic regression analysis yielded significant association levels of alcohol use and cigarette smoking, e-cigarette use and concurrent use of both, demonstrating the highest odds ratios, compared with any variable analyzed for this study, for each category of alcohol use, even at the lowest level of 1 or 2 days (Table 3). The odds ratios for concurrent cigarette and e-cigarette use were larger across all categories of alcohol use than for cigarette smoking and e-cigarette use alone. Odds ratios for 1 or 2 days of drinking were similar for cigarettes (AOR = 9.79; 95% CI, 9.08–10.56) and e-cigarettes (AOR = 9.25; CI 95% 8.78, 9.75) but higher for concurrent cigarette and e-cigarette use (AOR = 11.3; 95% CI 10.3–12.4). Odds ratios increased more steeply at higher levels of alcohol use for cigarette smoking than for e-cigarette use. 

## Discussion

Although the potential health harms of e-cigarettes are under study and yet largely unknown (3), investigation into patterns of e-cigarette use among adolescents, particularly when patterns diverge from those of cigarette smoking among adolescents, is an important priority for public health agencies. The Centers for Disease Control and Prevention and other public health agencies consider e-cigarette use among adolescents a public health concern (5,6), and one goal of Healthy People 2020 is the reduction of tobacco use among adolescents (11). Additionally, although e-cigarettes contain no tobacco, they are regulated by the US Food and Drug Administration as tobacco products because of their nicotine content (12).

This study adds to research (3) highlighting significant differences in psychosocial and behavioral risk factors predicting cigarette smoking and e-cigarette use among adolescents. This analysis showed significant associations between independent variables (sexual identity, socioeconomic indicators, alcohol use, and academic performance) and the outcomes of cigarette smoking and e-cigarette use. It also suggests that differences exist in the magnitude of risk for cigarette smoking or e-cigarette use for some categories of sexual identity, economic status, school performance, and alcohol use. For example, bisexual students, students reporting mostly Fs, and students reporting alcohol use 10 or more days in the past 30 days were at least twice as likely to smoke cigarettes as e-cigarettes. Further analysis should test these differences. Also of interest was that although in this sample of adolescents in Minnesota the prevalence of cigarette smoking (4.6%) was approximately half that of e-cigarette use (9.5%), the odds ratios for cigarette smoking were greater than for e-cigarette use across all categories of the independent variables analyzed. Ideally, further research will shed more light on the various risk factors for cigarette smoking and e-cigarette use among adolescents as well as on the health risks of e-cigarette use among this population. Such research would allow public health practitioners to more effectively target tobacco-reduction interventions by differentiating between cigarette smokers and e-cigarette users and determining which group may be at higher risk for harmful health outcomes.

This study has numerous limitations and opportunities for further analysis. One limitation was the extent of missing data. Missing data for demographic, independent, and outcome variables ranged from less than 1% for the question on sex (n = 373) to 6.9% (n = 8,760) for using e-cigarettes and 7.0% (n = 8,850) for cigarette smoking. For a sample size of 126,868, these are relatively small amounts. However, when comparing these numbers to the numbers of students who reported smoking (n = 5,816 or 4.6%) or using e-cigarettes (n = 12,101 or 9.5%), the extent of missing data is more relevant. Additionally, given that Minnesota is home to an estimated 40,000 people of Somali origin (13), 70,000 people of Hmong ethnicity (13), and 288,000 people of Hispanic or Latino ethnicity (13), having further insight into patterns of tobacco use among adolescents in these populations would be valuable to public health practitioners. If time had permitted, I would have conducted a more in-depth and systematic investigation into the reasons for missing data and how to account for them.

Another limitation of this study is use of a single cross-sectional survey. Given further time and a more robust study design, it would be worth conducting regression analyses separately across multiple years of the MSS. One opportunity for a more robust analysis of the MSS data set would be an investigation into the increased prevalence of cigarette smoking and e-cigarette use with each successive grade level. This investigation would include running separate regression analyses by grade rather than controlling for grade as well as developing an all-inclusive regression model for all variables.

One strength of this study is having used the MSS. Although studies of data from the National Youth Tobacco Survey (14) and the Minnesota Youth Tobacco Survey (15) provide a more robust inquiry into the home, environment, and exposure characteristics of adolescent smokers, both surveys have smaller sample sizes (20,675 in 2016 and 4,243 in 2014, respectively) that limit utility of extracting data on racial/ethnic and geographic characteristics (16,17). The larger sample size of the MSS potentially allows for a more robust analysis of racial/ethnic and geographic characteristics and an inquiry into a broader range of socioeconomic and behaviors.

This study adds to research indicating that socioeconomic and behavioral risk factors differ between students who smoke cigarettes and students who use e-cigarettes, further suggesting that public health outreach programs to reduce tobacco use among adolescents may need to differ in methods and messages, with program choices depending on the risk factors of the target audience and whether the goal is to reduce cigarette smoking or reduce e-cigarette use.
